# Genetic Variation in the Proximal Promoter of ABC and SLC Superfamilies: Liver and Kidney Specific Expression and Promoter Activity Predict Variation

**DOI:** 10.1371/journal.pone.0006942

**Published:** 2009-09-09

**Authors:** Stephanie E. Hesselson, Pär Matsson, James E. Shima, Hisayo Fukushima, Sook Wah Yee, Yuya Kobayashi, Jason M. Gow, Connie Ha, Benjamin Ma, Annie Poon, Susan J. Johns, Doug Stryke, Richard A. Castro, Harunobu Tahara, Ji Ha Choi, Ligong Chen, Nicolas Picard, Elin Sjödin, Maarke J. E. Roelofs, Thomas E. Ferrin, Richard Myers, Deanna L. Kroetz, Pui-Yan Kwok, Kathleen M. Giacomini

**Affiliations:** 1 Cardiovascular Research Institute, University of California San Francisco, San Francisco, California, United States of America; 2 Department of Bioengineering and Therapeutic Sciences, University of California San Francisco, San Francisco, California, United States of America; 3 Pharmaceutical Chemistry, University of California San Francisco, San Francisco, California, United States of America; 4 Department of Genetics, Stanford University School of Medicine, Stanford, California, United States of America; Dr. Margarete Fischer-Bosch Institute of Clinical Pharmacology, Germany

## Abstract

Membrane transporters play crucial roles in the cellular uptake and efflux of an array of small molecules including nutrients, environmental toxins, and many clinically used drugs. We hypothesized that common genetic variation in the proximal promoter regions of transporter genes contribute to observed variation in drug response. A total of 579 polymorphisms were identified in the proximal promoters (−250 to +50 bp) and flanking 5′ sequence of 107 transporters in the ATP Binding Cassette (ABC) and Solute Carrier (SLC) superfamilies in 272 DNA samples from ethnically diverse populations. Many transporter promoters contained multiple common polymorphisms. Using a sliding window analysis, we observed that, on average, nucleotide diversity (π) was lowest at approximately 300 bp upstream of the transcription start site, suggesting that this region may harbor important functional elements. The proximal promoters of transporters that were highly expressed in the liver had greater nucleotide diversity than those that were highly expressed in the kidney consistent with greater negative selective pressure on the promoters of kidney transporters. Twenty-one promoters were evaluated for activity using reporter assays. Greater nucleotide diversity was observed in promoters with strong activity compared to promoters with weak activity, suggesting that weak promoters are under more negative selective pressure than promoters with high activity. Collectively, these results suggest that the proximal promoter region of membrane transporters is rich in variation and that variants in these regions may play a role in interindividual variation in drug disposition and response.

## Introduction

Membrane transporters facilitate the uptake and efflux of endogenous compounds, ions, and drugs across cellular membranes. Two major superfamilies, the ATP binding cassette (ABC) and solute carrier (SLC) families, are recognized as important for the transport of drugs and other xenobiotics. ABC transporters are efflux pumps that rely on ATP hydrolysis to actively move substrates across biological membranes [1], [2]. In the ABC transporter superfamily, drug transport has primarily been associated with P-glycoprotein (*ABCB1*), Breast Cancer Resistance Protein (*BCRP/ABCG2*), and several members of the Multidrug-Resistance Associated Protein (MRP/ABCC) family. These transporters act to limit the access of drugs to protected tissue compartments, and to eliminate drugs and metabolites via bile and urine [3], [4]. Members of the SLC superfamily generally mediate the cellular uptake of nutrients such as glucose and amino acids, either through a facilitative transport mechanism where the substrate is translocated down its concentration gradient, or through secondary active transport mechanisms, where substrate translocation against a concentration gradient is coupled to ion flux along the cell membrane electrochemical gradient [5]. Like the ABC transporters, SLC transporters from a number of families accept a variety of structurally diverse drugs as substrates [6].

Variation at transporter gene loci may be responsible for differences in the therapeutic and adverse responses to pharmaceuticals [7]–[9]. The coding regions of many membrane transporters have been sequenced to locate polymorphisms that alter the structure and function of transporter proteins. As expected, the number and minor allele frequencies of non-synonymous polymorphisms in membrane transporters exons are generally small [10]. In contrast, in non-coding regions, polymorphisms are more abundant and minor allele frequencies are higher due to the lower selection pressure on these regions. Because of the generally lower genetic constraint in the regulatory regions of membrane transporters, we hypothesize that these regions may contain most of the common variation that underlies variable drug response. The proximal promoter has been defined as the region immediately surrounding the transcriptional start site, which is critical in binding polymerase complexes necessary for gene transcription [11], [12]. Polymorphisms in the proximal promoter may alter the rate of transcription resulting in changes in gene expression, thereby ultimately affecting the level of cellular uptake or efflux. Polymorphisms that alter the expression levels of membrane transporters could determine the effective dosage of a drug by altering the systemic or tissue specific exposure to the drug. Some polymorphisms in the proximal promoter of membrane transporter genes identified in this sequencing effort have been shown to alter transcription rates in reporter assays and associate with expression levels in human lymphoblastoid cell lines [13], [14].

To facilitate the discovery of variants that may influence drug transporter expression levels, we sequenced the proximal promoters (−250 bp upstream to 50 bp downstream of the transcription start site) [15] of 107 transporters including 42 ABC and 65 SLC membrane transporter genes (Table S1). To identify both common and rare variants we used a large sample consisting of DNA from 272 individuals from four populations. The level of variation in the proximal promoters of membrane transporters was compared to other gene regions. Using population genetic parameters, we compared variation among transporter families and superfamilies, and in transporters expressed primarily in the liver versus those expressed primarily in the kidney. We experimentally determined promoter activity using reporter assays and compared variation in strong and weak promoters. These data will contribute to the characterization of non-coding polymorphisms on the gene expression of membrane transporters.

## Results

### Polymorphism Discovery

#### New polymorphisms identified

A total of 52,445 base pairs were sequenced, and 579 polymorphisms (out of which 207 were singletons) were observed (Table 1). Single nucleotide polymorphisms (SNPs) were the most common type of polymorphism (93.6%). Cross-reference of our variants to the dbSNP database indicated that 369 polymorphisms were novel (dbSNP build 129) (Table S2). Of these novel variants, 54 had minor allele frequencies greater than 5% in at least one population.

**Table 1:** Summary of variation in membrane transporter promoters.

		Base Pairs		SNPs	MAF <1%	SNPs	MAF≥1%	Indels		Total
	n	PP	5′	PP	5′	PP	5′	PP	5′	Total
All Genes	107	31715	20730	133	74	214	121	26	11	579
All ABC	42	12464	9952	45	17	65	48	8	6	189
ABCA	9	2667	2140	13	2	13	7	3	1	39
ABCB	10	2987	1895	5	2	19	7	3	2	38
ABCC	11	3251	3054	12	6	12	14	1	2	47
ABCG	5	1500	1215	8	5	8	11	1	1	34
Other ABC	7	2059	1648	7	2	13	9	0	0	31
All SLC	65	19251	10778	88	57	149	73	18	5	390
SLC6	13	3889	2900	17	14	40	19	1	0	91
SLC17	6	1713	1074	9	9	9	2	0	2	31
SLC22	17	5037	1983	24	9	33	7	6	0	79
SLC28/29	6	1800	1238	10	2	15	12	1	0	40
SLCO	7	2100	945	8	4	11	3	3	2	31
Other SLC	16	4712	2638	20	19	41	30	7	1	118

MAF: Minor allele frequency, PP: proximal promoter.

The sequenced range covered 31,715 base pairs in proximal promoters and 20,730 base pairs of non-coding sequence upstream of the proximal promoters. In total, 373 polymorphisms were observed in the proximal promoters and 206 in the 5′ flanking regions. The observed frequency of polymorphisms in the 5′ flanking regions (1/101 bp) was thus comparable to our previous findings in non-coding intronic regions in 24 transporters (1/118 bp) [10]. The proximal promoter regions (1/85) were more polymorphic than the intronic and 5′ flanking regions (Table 1).

#### Allele frequencies of polymorphisms

**Figure 1:**
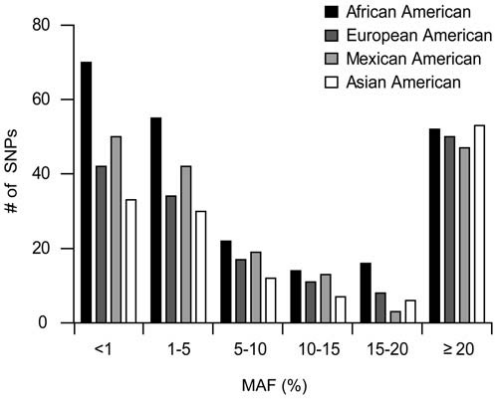
Ethnic breakdown of proximal promoter SNPs. The number of SNPs present in each ethnic group are represented by shaded bars: African Americans (black), European Americans (dark grey), Mexican Americans (light grey), and Asian Americans (white). SNPs are categorized based on the minor allele frequency (MAF) occurring in each corresponding ethnic group.

The number of polymorphisms per proximal promoter ranged from zero to fourteen, with the majority of transporters (90%) having at least one common (MAF≥5%) polymorphism or multiple singletons in this region. Only 10% of promoters had no polymorphisms or only one singleton. The maximum number of singletons in any promoter was four. Common polymorphisms with MAF>5% in at least one population were found in 66% of proximal promoters. Interestingly, a large number of polymorphisms were found at a frequency of 20% or greater (Figure 1), which is in distinct contrast to polymorphisms found in coding regions of membrane transporters [10]. Transporters with the highest number of polymorphisms in their proximal promoter were *SLC7A5*, which transports large neutral amino acids; *SLC47A1*, which transports endogenous and exogenous organic cations; *ABCC5*, which exports cyclic nucleotides; *ABCD4*, involved in the transport of fatty acids; *SLC22A5* and *SLC22A16*, organic anion/cation transporters; *SLC29A3*, involved in the uptake of nucleotides, anti-cancer and anti-viral drugs; *SLC17A3*, a phosphate transporter located in the endoplasmic reticulum membrane; *SLC15A1*, involved in the intestinal absorption and renal disposition of di- and tripeptides; and the sodium neurotransmitter symporters *SLC6A4*, *SLC6A12*, *SLC6A17*, and *SLC6A19* (Table S3).

#### Population specificity of polymorphisms

The expected distributions of polymorphisms were observed in the four examined populations. In particular, a larger number of singletons and low frequency polymorphisms were observed in the African American population than in the other three populations (Figure 1) and the overall number of polymorphisms in African Americans was also higher than for any other population. However, 36% of the polymorphisms were not observed in the African American population and thus the addition of multiple populations contributed greatly to the magnitude of variation identified in the total sample (Table 2).

**Table 2:** Proximal promoter SNPs shared among 4 major ethnic groups.

Ethnic Groups					
AA	EA	ME	AS	# of SNPs	% of Total SNPs
x				90	26.0
	x			30	8.6
		x		33	9.5
			x	42	12.1
x	x			7	2.0
x		x		17	4.9
x			x	2	0.6
	x	x		8	2.3
	x		x	5	1.4
		x	x	5	1.4
x	x	x		24	6.9
x	x		x	4	1.2
	x	x	x	1	0.3
x		x	x	1	0.3
x	x	x	x	78	22.5
223	157	167	138	347	

AA: African American; EA: European American; ME: Mexican American; AS: Asian American.

#### Insertions and Deletions

Thirty-seven of the polymorphisms identified were insertions or deletions (indels). The ratio of 37 indels to 537 SNPs (ca. 0.07) was similar to what was previously observed in intronic regions of ABC and SLC transporters (21 indels/350 SNPs or 0.06) [10]. Importantly the indel to SNP ratio in promoter regions was substantially greater than that observed in coding regions of ABC and SLC transporters (8 indels/330 SNPs or 0.02). These results suggest that insertion and deletion events, which may involve genomic rearrangement and recombination, are more likely in the upstream region than in the coding regions of genes. This observation is expected because indels in coding regions can lead to frameshift mutations, which result in a truncated protein that may be hyper-, hypo-, or neomophic explaining the high negative selective pressure in coding regions.

A greater number of indels per bp sequenced was observed in the proximal promoters (1/1220) than in the 5′ flanking regions (1/1885 bp). The type of indel also differed between proximal promoters and the 5′ flanking sequence. Single base pair indels made up a greater percentage of the total indels in the 5′ flanking regions (91%) than in the proximal promoters (27%). It has been reported that a majority of indel sequences in the human genome are AT rich [16]. However, in the proximal promoters in our dataset, a majority (62%) of the indel sequences were instead GC rich. This is consistent with the observation that a large proportion of mammalian promoters contain CpG islands [12], [17]. Most indels identified in this sequencing effort were flanked by identical sequence. This was true for both single bp indels that have at least one base pair of the same identity next to the insertion or deletion site (91%), and for multibase indels that have the identical complex sequence flanking the indel (54%). This leads to ambiguity in the exact location of the indel. The flanking sequence for all indels can be viewed at http://pharmacogenetics.ucsf.edu/ and Table S2).

#### Haplotypes

Strong linkage disequilibrium between polymorphisms in the 300 bp proximal promoter region was observed in all populations, as would be expected due to their proximity (Table S4). In general, when the minor alleles of polymorphisms were present in multiple populations, they also formed the same haplotypes in all populations. In most cases the minor allele of each polymorphism was in a haplotype that did not include the minor allele of any other polymorphism. Unexpectedly there was more haplotype diversity in *SLC22A5* in the Chinese population than in any other population. The Chinese samples had four common haplotypes that differed from the reference haplotype, whereas the other three populations had only two haplotypes with frequencies above 2%. This may be related to a Chinese specific fixation of the minor allele of a SNP just downstream of the proximal promoter, which is present on all four common haplotypes.

#### Polymorphisms in ABC and SLC superfamilies

The two transporter superfamilies differed in the amount of genetic diversity observed. More polymorphisms were observed in the SLC promoters than in the ABC promoters, with both common and rare polymorphisms contributing to this observation. (Table 1) On average a polymorphism occurred every 106 base pairs in ABC transporter proximal promoters and every 75 base pairs in SLC transporters. Correspondingly, the fraction of transporters that had no polymorphisms or only one singleton in the proximal promoter was greater in the ABC (14%) than in the SLC superfamily (8%). This trend was also observed for polymorphisms with minor allele frequencies greater than 5%. That is, a lower percentage of ABC promoters (57%) had at least one polymorphism with a MAF>5% in at least one population compared to SLC promoters (72%).

### Population Genetics of Transporter Promoters

#### Population genetic statistics

Genetic variation in exons is constrained by negative selection [10], [18], [19]. To determine if this was also the case for the proximal promoter regions we calculated the population genetic parameters π (nucleotide diversity/average heterozygosity) and θ (the population mutation parameter) for each promoter, as well as two estimates of selection pressure: Tajima's D and Fay and Wu's H statistics (Tables 3, 4 and S3). Proximal promoters in membrane transporters appear to be characterized by greater genetic variation than coding regions of these genes. In particular, a θ of 15 and a π of 9.6 were calculated for 102 proximal promoters (Table 3). These values are greater than the values of 8.9 and 4.0 observed previously for θ and π in the coding region of 24 membrane transporters [10]. These data suggest that variations in promoters are less deleterious than in exons, which have much less heterozygosity. Consistent with the greater number of polymorphisms observed in the SLC than in the ABC family, the values of π and θ in both the proximal promoters and 5′ upstream flanking sequence were higher in SLC transporters (Table 3). Values of π were also higher in many protein domains in the SLC transporters than in the ABC transporters [10]. These data suggest that in general, SLC transporters can tolerate more variation than ABC transporters, possibly due to functional redundancies within many SLC subfamilies. The trends for π and θ were similar in all of the populations (Table 3).

**Table 3:** Summary of population genetics statistics of membrane transporter promoters.

		θ	x	10^4^	θ	x	10^4^	θ	x	10^4^	π	x	10^4^	π	x	10^4^	π	x	10^4^
	Pop.	PP			5′			Total			PP			5′			Total		
All Genes	AA	12.1	±	2.9	9.2	±	2.4	11.0	±	2.6	10.1	±	4.9	6.9	±	3.5	8.9	±	4.3
(n = 102)	EA	8.2	±	2.0	5.4	±	1.5	7.2	±	1.8	8.8	±	4.3	4.9	±	2.5	7.3	±	3.6
	ME	9.3	±	2.3	6.8	±	1.8	8.4	±	2.0	8.4	±	4.1	5.0	±	2.6	7.1	±	3.5
	AS	7.6	±	1.9	5.6	±	1.5	6.8	±	1.7	8.2	±	4.0	5.2	±	2.7	7.1	±	3.4
	Total	15.3	±	3.0	11.4	±	2.3	13.9	±	2.7	9.6	±	4.6	5.9	±	3.0	8.2	±	4.0
All ABC	AA	10.1	±	2.6	7.5	±	2.1	8.9	±	2.3	9.1	±	4.6	5.5	±	3.0	7.5	±	3.7
(n = 41)	EA	6.3	±	1.8	3.5	±	1.1	5.0	±	1.3	7.8	±	4.0	3.7	±	2.1	6.0	±	3.0
	ME	8.2	±	2.2	4.8	±	1.5	6.7	±	1.7	7.9	±	4.0	3.7	±	2.1	6.0	±	3.0
	AS	7.0	±	1.9	4.0	±	1.3	5.7	±	1.5	7.7	±	3.9	3.6	±	2.0	5.9	±	2.9
	Total	12.8	±	2.7	8.0	±	1.8	10.7	±	2.2	8.8	±	4.4	4.4	±	2.4	6.8	±	3.4
All SLC	AA	13.4	±	3.3	11.3	±	3.1	12.7	±	3.1	10.8	±	5.3	8.7	±	4.5	10.1	±	4.9
(n = 61)	EA	9.6	±	2.4	7.7	±	2.2	9.0	±	2.2	9.5	±	4.7	6.4	±	3.4	8.5	±	4.2
	ME	10.1	±	2.6	9.3	±	2.6	9.8	±	2.4	8.8	±	4.4	6.5	±	3.5	8.1	±	4.0
	AS	8.0	±	2.1	7.5	±	2.2	7.8	±	2.0	8.5	±	4.2	7.2	±	3.8	8.1	±	4.0
	Total	17.0	±	3.4	15.5	±	3.3	16.5	±	3.2	10.1	±	5.0	7.7	±	4.0	9.4	±	4.6

AA: African American; EA: European American; ME: Mexican American; AS: Asian American. Pop: population, PP: proximal promoter.

**Table 4:** Positive selection in membrane transporter promoters.

		H (Significance)			
		AA	EA	ME	AS
ABC Transporters	Significant in		4 Populations		
	ABCD2	−3.56(**)	−3.79(***)	−3.86(***)	−3.81(***)
	ABCD4	−5.09(*)	−5.52 (**)	−5.21(*)	−5.54(***)
	Significant in		3 Populations		
	ABCB9	−0.76*(n. s.)*	−2.31(***)	−2.10(*)	−2.73(**)
	ABCC3	−0.89*(n. s.)*	−1.59(*)	−1.58(*)	−3.44(**)
	ABCC9	−3.87(***)	−3.97(***)	−4.01(***)	*n. a.*
	Significant in		2 Populations		
	ABCA4	−0.66*(n. s.)*	*n. a.*	−1.86(*)	−1.97(**)
	ABCD1	0.22*(n. s.)*	−1.55(*)	−1.35(*)	−1.34*(n. s.)*
Total *n* transporters with significant H		3	6	7	5
SLC Transporters	Significant in		4 Populations		
	SLC19A2	−1.97(*)	−2.76(**)	−2.49(**)	−1.81(*)
	SLCO1A2	−5.72(***)	−5.84(***)	−5.08(**)	−5.00(***)
	SLC22A10	−1.74(*)	−1.87(*)	−1.90(*)	−2.00(***)
	Significant in		3 Populations		
	SLC6A4	−1.85(**)	−1.81*(n. s.)*	−1.82(*)	−1.91(*)
	SLC6A14	−0.51*(n. s.)*	−1.62(*)	−1.59(*)	−1.94(*)
	SLC47A1	−2.00(*)	−1.93*(n. s.)*	−2.01(*)	−2.01(*)
	SLCO1B1	−1.74*(n. s.)*	−1.90(*)	−1.97(*)	−1.90(*)
	Significant in		2 Populations		
	SLC6A12	−1.60*(n. s.)*	−1.81(*)	−1.66*(n. s.)*	−2.00(***)
	SLC15A1	−1.20*(n. s.)*	−1.94(*)	*n. a.*	−1.93(**)
	Significant in		1 Population		
	SLC18A1	−0.96*(n. s.)*	0.04*(n. s.)*	−0.41*(n. s.)*	−1.23(*)
	SLC22A12	−1.51*(n. s.)*	−2.67(*)	−2.20*(n. s.)*	−1.53*(n. s.)*
Total *n* transporters with significant H		5	8	7	10

#### Regional differences in nucleotide diversity

**Figure 2:**
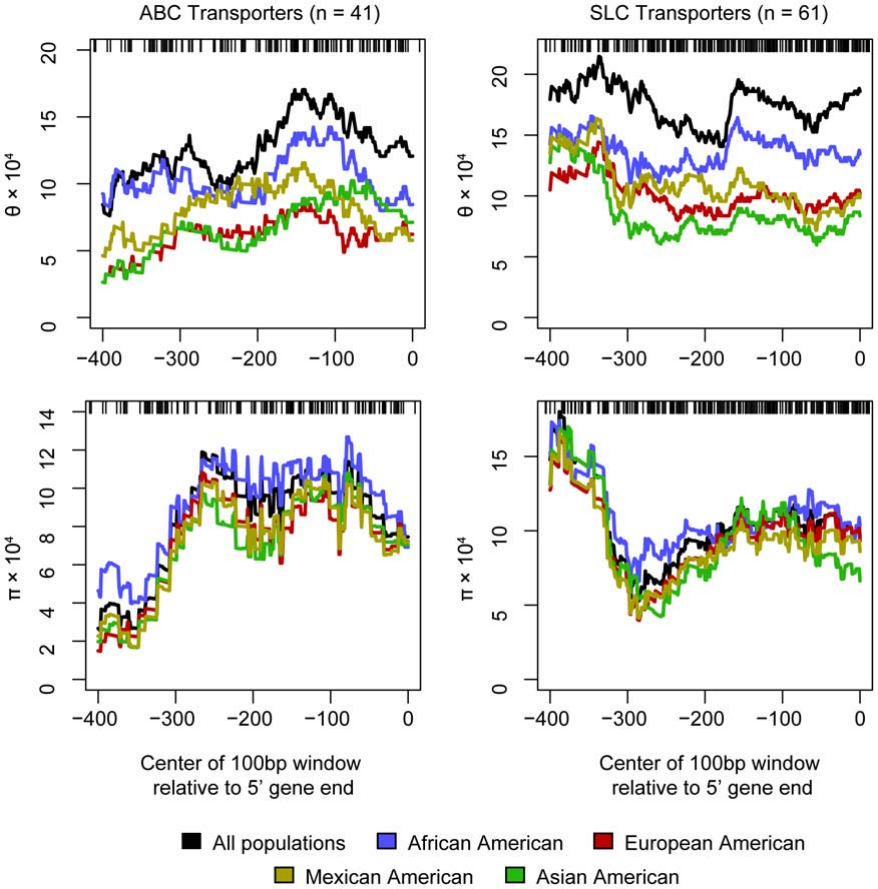
Location of nucleotide diversity in transporter promoters relative to the transcription start site. Nucleotide diversity was estimated using the mutation parameter (θ) and the average heterozygosity per site parameter (π), in a 100 bp sliding window. The diversity parameters are plotted as functions of the center of the sliding window. Blue line: African Americans; red line: European Americans; gold line: Mexican Americans; green line: Asian Americans; and black line: combined population.

On average, more variation was observed in the proximal promoter regions than in the 5′ flanking sequence (Table 1 and 3). To get a more detailed view of the regional differences, we used a sliding window approach to determine local nucleotide diversity in ABC and SLC transporters in each of the examined populations (Fig. 2). In the ABC transporters, both θ and π were at a maximum in a range centered around 150 bp upstream from the transcription start site. A similar peak in nucleotide diversity was also observed in the SLC transporters, but in contrast to the ABCs, these transporters also showed a sharply increased variability further upstream. Common to both families was a minimum diversity centered around 250 bp upstream from the transcription start site.

#### Positive and negative selection

Though variation in most promoters was consistent with the infinite sites neutral model [20], some ABC and SLC transporters showed signs of positive selection (Table 4). The observation of positive selection is consistent with previous findings of positive selection in promoters of other genes [21], [22]. The proximal promoters of many transporters appear to have similar pressures acting on them in more than one population. *ABCD2* and *ABCD4* both involved in the transport of fatty acids, and *SLCO1A2*, which mediates cellular uptake of organic ions such as bile acids and steroidal compounds, appeared to have the greatest degree of positive selection as indicated by the low H values in all four populations (Table 4). Other transporters that showed some degree of positive selection in several populations include *SLC19A2*, a vitamin B1 transporter, the sodium neurotransmitter symporters *SLC6A4* and *SLC6A14*, and some ABC transporters involved in multidrug resistance, (*ABCB9*, *ABCC3* and *ABCC9*). In contrast to these data showing positive selection, *ABCA13*, which encodes the largest ABC transporter with 5,058 amino acids [23], had significantly positive H values in two populations consistent with purifying or negative selection (Table S3). All other promoters were consistent with the infinite sites, neutral model.

### Relationship Between Genetic Variation and Promoter Activity

#### Variation in hepatic and renal transporters

**Figure 3:**
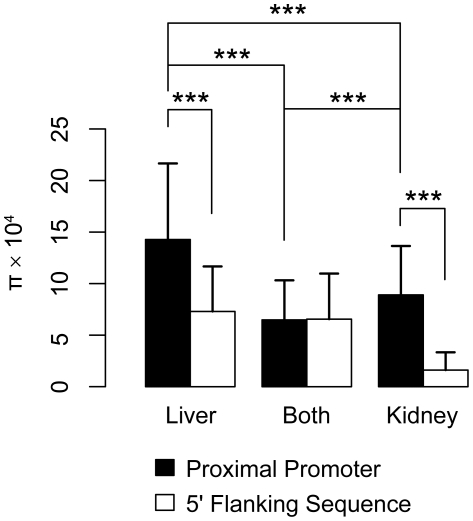
Nucleotide diversity in transporters expressed in human liver and/or kidney. Transporters were included in the analysis if expressed in the top one-third of all assessed probes in mRNA microarray experiments, or if significantly expressed in real time PCR experiments (See Materials and Methods). A list of the transporters is provided as Table S5. Nucleotide diversity is shown for the proximal promoter (black bars) and the 5′ flanking region (open bars).

Because many of the transporters analyzed in this study play a role in hepatic and renal drug elimination, we compared genetic variation in liver and kidney transporters. Transporters were classified as having significant expression in liver or kidney (or both) if their expression levels ranked in the top third of expressed genes in multiple sources of microarray data [24]–[27] or they showed significant expression in RT-PCR experiments [28]. The transporters classified as being expressed in the kidney, the liver, or in both tissues based on these available datasets are presented in Table S5. We then determined the nucleotide diversity for the transporters included in each of the three groups. Transporters that were predominantly expressed in the liver were found to have higher average heterozygosity than transporters that were highly expressed in the kidney, and transporters expressed at high levels in both tissues had the lowest average heterozygosity (Figure 3 and Table S6).

### Genetic variation in proximal promoters expressed in cell lines

**Figure 4:**
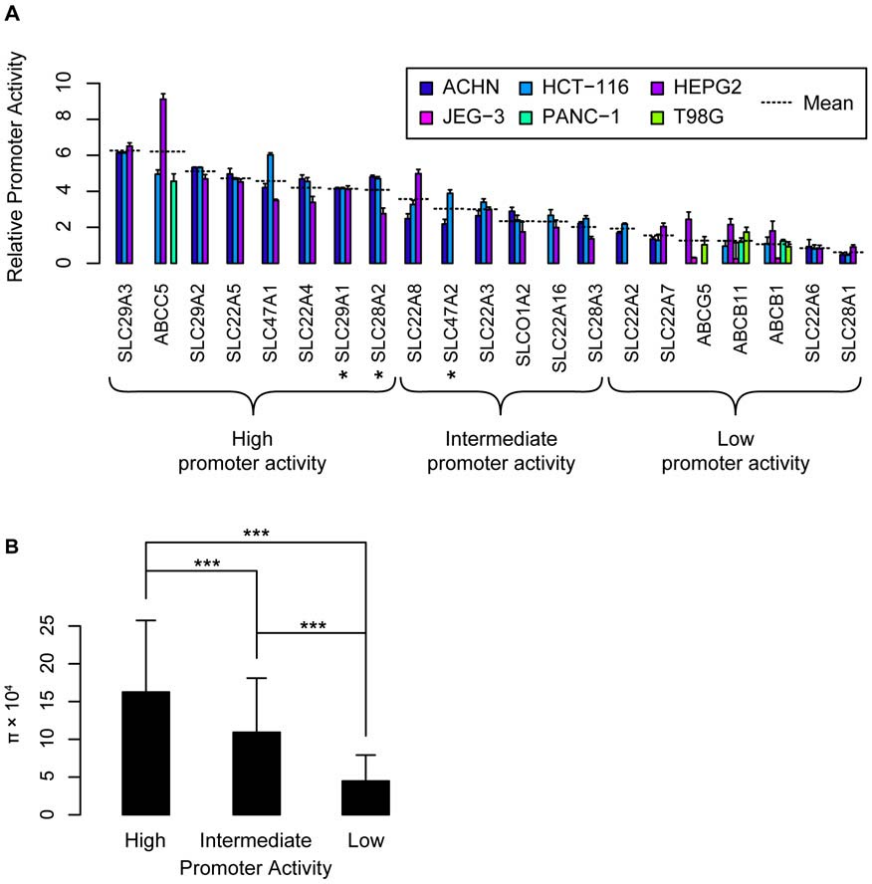
A. Reporter activity of proximal promoter constructs in human cell lines. The proximal promoters were amplified from human genomic DNA and cloned into pGL-4 gene expression vectors. Luciferase activity was measured 24 h after transfection of a panel of human cell lines. Promoter activity is expressed as *log_2_ [(1+ Firefly luciferase/Renilla luciferase)/Average of the negative control]*. Promoters were classified as having high, intermediate or low activity if the average relative activity across multiple cell lines was above 4, between 2 and 4, or below 2, respectively. B. Nucleotide diversity in high, intermediate and low activity promoters.

To confirm that the sequenced regions contain a functional promoter, we cloned a subset of the sequenced regions into firefly luciferase reporter constructs and expressed them in continuous cell lines from gastrointestinal epithelia (JEG-3 and HCT-116), kidney (ACHN), liver (HEPG2), pancreas (PANC-1) and glioblastoma (T98G). We selected 21 promoters of the two major superfamilies, SLC and ABC membrane transporters that play significant role in transporting drug molecules and xenobiotics into or out of the cells. In addition, these selected groups of transporters, such as organic cation transporters (SLC22A), nucleoside transporters (SLC28A and SLC29A) and ABC transporters have high expressions in liver and/or kidney tissues where they play important roles in drugs/xenobiotics absorption, distribution and elimination. Transcriptional activity of the most common haplotype of each promoter is shown in Figure 4A. The mean activity across all tested cell lines was significantly above the negative control in 15 out of 21 promoters suggesting that the majority of the cloned regions have measurable promoter activity (Figure 4A). We designated promoters as having low, intermediate or high activity based on the observed change in luciferase activity compared to negative controls and we observed that genetic variation was greater in promoters with the greatest activity. In particular, the third of promoters with the highest activity (>5 fold above the control vector) had significantly greater π values than the third of promoters with the lowest activity (<2 fold above the control vector) (Figure 4B). Intermediate activity promoters had intermediate π values. These results were also mirrored in a greater average number of polymorphisms per promoter (5) for the promoters with high activity than for those with low activity (2.7).

SNPs in the proximal promoter may alter expression levels of the transporters by affecting the rate of transcription. We used publicly available data for gene expression in immortalized lymphoblastoid cell lines derived from the individuals in the HapMap cohort [29], and correlated these with genotype data for the same individuals. Notably, out of a total of 46 common (MAF>5%) SNPs that were observed both in our study cohort and in the populations sampled in the HapMap project, five SNPs were significantly associated with altered levels of transporter expression in lymphoblastoid cell lines (Table S7). This resulted in a significantly higher frequency (11% of the interrogated SNPs) compared to the frequency of associations at the same significance level when all HapMap SNPs in a ±50,000 bp range surrounding the same transporters was considered (3%).

## Discussion

### Proximal promoters of membrane transporter genes have a high degree of variation

A strength of this study compared to genome-wide genotyping efforts such as the HapMap project, is that the complete resequencing of 107 membrane transporter promoters allowed polymorphism discovery that was unbiased by previous knowledge. In contrast, the HapMap project aimed to characterize the entire human genome, at the expense of having lower coverage of individual regions [30]. We observed a high degree of genetic variation in the upstream regions of membrane transporter genes including both the proximal promoters (−250 bp to +50 bp) and the 5′ flanking regions. Based upon population genetic analysis, this variation was higher than variation reported previously in the coding region or flanking intronic regions of a subset of these membrane transporter genes [10]. It might be expected that the loci that contain a proximal promoter would be less likely to accumulate polymorphisms and for polymorphisms in this region to have relatively low minor allele frequencies due to functional constraint. However, many membrane transporter promoters not only have a high number of polymorphisms, but many of these polymorphisms also have high minor allele frequencies. One explanation for this is that the actual proximal promoter, which binds the transcriptional machinery, may be small and may vary depending upon the gene. For example, in 45 human genes, Cooper et al observed that, on average, the maximal promoter activity assessed in reporter assays occurred between −250 and −350 bp upstream of the transcriptional start site [11]. This region may represent a true basal or proximal promoter. Notably, this region coincides with a region of low variation in the sliding window analysis of π and θ in our study, which may contain important functional sites (Figure 2). An alternative explanation is that polymorphisms in regulatory regions may be altering the expression of genes and that this altered expression may be contributing to human evolution in some cases [31].

### Promoter polymorphisms may alter gene expression

Polymorphisms in cis-regulatory regions have been shown to alter phenotypic traits in many species including humans. Some examples in humans include malaria resistance and lactase persistence [31]. SNPs in the promoters of transporters have been shown to associate with altered expression levels. For example, Poonkuzhali et al described novel SNPs in the *ABCG2* promoter region (-15994C>T (rs7699188), -56846A>C, and -15622C>T) that were significantly associated with *ABCG2* expression in lymphoblast, liver and/or intestine tissues, and one of these was moderately associated with clearance of the anticancer drug, imatinib [32]. We have also identified SNPs in this study that cause altered promoter activity in reporter assays. For example, the nucleoside transporter, *SLC28A2*, has five SNPs in its proximal promoter, and the minor allele of one of these (rs2413775 MAF≥20% in all four populations) was associated with increased luciferase activity in cell lines and in *in vivo* injections of the reporter construct into mouse liver [13]. The carnitine transporter, *SLC22A5*, has six SNPs in its proximal promoter. The G allele of rs2631367 is associated with higher luciferase activity and expression levels in lymphoblastoid cell lines. The G allele is monomorphic in Asians and has an allele frequency between 50–60% in the other three populations. This allele contributes to observed ethnic differences in expression level of the transporter in lymphoblastoid cell lines [14]. Additional polymorphisms observed in this study may regulate expression levels of transporters and could provide an explanation for some variability observed in drug disposition.

### Some proximal promoters of membrane transporters show signs of positive selection

The signature of positive and negative selection is the high number of rare variants as opposed to common ones [33]. Many mutations in loci experiencing negative selection, such as coding regions, do not increase in frequency because they do not increase fitness. In contrast, for loci experiencing positive selection, new mutations do increase fitness and rise in frequency until the ancestral allele is reduced to a low frequency. For most of the promoters in our study, there was no evidence for either positive or negative selection, as assessed by both Tajima's D and Fay and Wu's H statistics (Table S3). However, for some promoters, the ancestral allele (defined as the allele shared between human and chimpanzee) was rare, indicating that there is a positive selection pressure on the derived, human-specific allele. These data suggest that mutations in the proximal promoters are often neutral and sometimes advantageous. This is in contrast to the coding regions of membrane transporters, which were previously shown to have low values of π and negative D values, indicative of negative selection pressure [10]. This suggests that new mutations in the promoter regions of membrane transporters are less likely to be disadvantageous than mutations in the coding regions.

The observation of positive selection in membrane transporter promoters is consistent with previous findings. Positive selection was observed in some proximal promoters in human specific and primate specific transcription factor binding sites in a genome wide analysis of Hap Map and Perlegen SNPs [21] and has also been found in genes involved in brain activity and in nutrient homeostasis [22]. In this study, of the promoters that had significant H values in at least two populations, several encoded transporters involved in neural function and nutrient disposition (e.g., sodium neurotransmitter symporters and proteins involved in the transport of fatty acids, vitamin B1, and cyclic nucleotides). One of the transporters, *SLC6A4*, encodes the serotonin transporter, *SERT*. This transporter is responsible for the re-uptake of serotonin into pre-synaptic neurons and is the target of many clinically used anti-depressants [34]. A promoter variant in this transporter found upstream of the region that we analyzed has been associated with many clinical phenotypes including depression and suicide [35]. Polymorphisms in the cis-regulatory regions of *SLC6A4* have also been associated with creativity in humans and dispersal behavioral in macaques [31]. Our finding that this and another SLC6 family neurotransmitter transporter is under positive selection is consistent with the rapid evolution of the human brain. It is also interesting that *SLCO1A2* (*OATP1A2*), the transporter whose promoter exhibited the highest degree of positive selection in all four populations, is found in the blood brain barrier [36]. This transporter is thought to play a role in the regulation of thyroid hormone levels in the brain and in the CNS levels of various other endogenous compounds and xenobiotics.

### Proximal promoters of liver transporters show greater variation than those of kidney transporters

We observed greater variation in the proximal promoters of transporters expressed primarily in the liver in comparison to those expressed primarily in the kidney. These results suggest that the promoter regions of transporters expressed primarily in the kidney are under more selective pressure than those of transporters expressed primarily in the liver. One explanation may be that many liver transporters are known to be highly inducible and thus are associated with a wide range of expression levels in human liver [37]. Selective pressure against variants in the proximal promoter region, which could potentially result in altered expression levels, may be low. In contrast, kidney transporters are generally not as inducible as liver transporters, and there may be more selective pressure against genetic variation in their promoter regions that result in variation in the expression levels of the transporters. High genetic variation in the promoter regions of liver transporters may thus contribute to the well-recognized wide inter-individual variation in hepatic drug clearance, which is much greater than variation in renal drug clearance [38]. Our results also suggest that there may be a greater number of redundant regulatory mechanisms that influence the rate of transcription of transporters expressed primarily in the liver than those expressed primarily in the kidney. While the proximal promoter regions examined in these liver expressed genes may be sufficient to control expression, they may not be the only promoter regions responsible for the overall control of hepatic expression levels. A majority of human genes have been shown to contain multiple alternative promoters [11], [12]. This has been proposed as a physiological mechanism to dynamically regulate expression levels of transporters and other genes depending on the tissue and on external stimuli [11], [12], [39], [40], and could allow for variation in the proximal promoter regions without significant detrimental changes in overall expression.

### Strong promoters show greater variation than weak promoters

One of the most interesting observations in this study was that promoters showing high activity in luciferase reporter gene assays had significantly more variation than promoters with intermediate or low activity (Figure 4B). Though speculative, it is possible that strong promoters may be able to tolerate mutations that result in moderately reduced or increased activity, because their transcriptional activity would still be sufficient to maintain functional expression levels. In contrast, weak promoters may not be able to tolerate mutations that result in reduced activity, since even minor decreases in promoter activity could result in deleteriously low transcriptional rates and expression levels of these genes. Although it cannot be ruled out that the weak promoters do not represent the true proximal promoters of these genes due to prediction errors, we deem it unlikely that random prediction errors would result in the trend seen in Figure 4B. It is also possible that the weak promoters may rely on additional enhancer elements upstream of the regions cloned into the expression system. Transcription factor binding sites and DNA motifs that direct tissue specific expression are often found further upstream than 250 bp from the transcription start site [41]. These regions may have comparable levels of genetic variation as the strong proximal promoters. Nevertheless our experimental results suggest that most of the loci that we sequenced do drive transcriptional activity in cell lines. The observed alleles can be tested for their effects on expression and eventually on drug response. Ultimately, investigation of the alleles discovered in this study may improve the safety and efficacy of commonly used pharmaceuticals.

### Conclusions

In summary, we resequenced the promoters of 107 membrane transporters in ethnically diverse populations. We detected a total of 579 polymorphisms, and 369 SNPs had not been reported previously. On average, variation was found to be higher in SLC than in ABC transporters, and in transporters highly expressed in the liver than in kidney-specific transporters. The function of a subset of the promoters was experimentally verified in a reporter system, and promoters with more genetic variation were shown to have higher activity than had less variable promoters regions.

## Materials and Methods

### Ethics Statement

Written informed consent was obtained from all individuals who provided their DNA for this study. The collection of these samples was approved by UCSF's Committee on Human Research (CHR approval # H531-17912-09B).

### Variant Identification

#### Study Subjects

Sequenced subjects are a subset of the SOPHIE cohort representing ethnically diverse populations in the San Francisco Bay Area. Subjects are healthy,18–40 year old males and females. Subjects have four grandparents from the population they identify with (African American, Caucasian, Chinese, and Mexican). Subjects were recruited from the Bay Area via flyers and on-line postings and several sites in San Francisco These sites include the Community Health Network of San Francisco, San Francisco General Hospital (SFGH), local churches, colleges and community organizations such as La Clinica de La Raza Oakland, Fair Oaks Clinic Redwood City, Health & Environment Resource Center (HERC) Bayview, Hunters Point, and SFGH GCRC. Subjects self-reported as healthy, non-smokers, non-excessive alcohol drinkers (<2 drinks/day), and are not on chronic medications. The SOPHIE cohort includes 194 African Americans, 150 Mexican Americans, 178 Chinese Americans, and 264 Caucasian. DNA was extracted from frozen blood samples and stored at −80°C until use.

#### Sequencing

Genomic DNA from 272 subjects was sequenced. Sixty-eight subjects each of African American (41 female/27 male), Caucasian (34 female/34 male), Chinese (44 female/24male), and Mexican (49 female/19 male) descent make up the sequenced panel. PCR primers were designed to include 250 bp upstream and 50 bp downstream of the transcriptional start site of the RefSeq gene listed in the UCSC database. Primers were designed using Primer 3 (http://frodo.wi.mit.edu/cgi-bin/primer3/primer3_www.cgi). There were two general methods for PCR. The first protocol used 4 ng genomic DNA in a10 µl reaction composed of 1 µl of 10Xbuffer, 0.7 µl of MgCl_2_(50 mM), 0.4 µl of dNTP (2.5 mM), 0.03 µl of Platinum taq (5 U/µl), 2 µl of Forward primer(1 µM), and 2 µl of Reverse primer(1 µM) with cycling conditions of 95°C for 2 min, 35 cycles of 92°C for 10 sec, 60°C for 20 sec, 72°C for 1 min, after the end of the 35 cycles followed by a 10 min hold at 72°C. The second protocol used 8 ng genomic DNA in 10 µl reaction composed of 1 µl of Buffer, 2 µl of Q-mix, 0.4 µl of dNTP (2.5 mM), 0.06 µl of Qiagen Taq polymerase, 2 µl of Forward primer (2 µM), 2 µl of Reverse primer (2 µM) (Buffer, Q-Mix, and enzyme are from Qiagen hot start kit) with cycling conditions of 95°C for 15 min, 35 cycles of 94°C for 1 min, 60°C for 1 min, 72°C for 1 min, after end of the 35 cycles followed by a 10 min hold at 72°C. PCR and sequencing primers in addition to annealing temperature are listed in Table S8. Purified PCR products were sequenced in one direction using ABI PRISM BigDye terminator sequencing Version 3.1 and an ABI Prism 3730 DNA analyzer. The 12 µl sequencing reaction was composed of 2.5 µl of ExoSapped PCR product, 4.5 µl of sequencing primer (1 µM), 1 µl BigDyeV3.1, 2 µl of 5X buffer, and 2 µl water. Cycling conditions were 96°C for 2 min, 25 cycles of 96°C for 15 sec, 50°C for 1 sec, 60°C for 4 minutes. DNA sequence files were imported into and scored with SEQUENCHER (Gene Codes, Ann Arbor, MI) Due to technical difficulties the following promoters were missing more than 20 bp of the target region: *ABCA3*, *ABCB5*, *ABCB6*, *ABCD2*, *SLC6A9*, *SLC9A3*, *SLC17A8*, *SLC22A14*, *SLC32A1*, *SLCO5A1*, and *SLCO6A1*.

#### Error Analysis

HWE tests were performed for each polymorphism within each population. Due to the large number of tests (1194) a HWE p-value cutoff of 0.01 was used. In most cases only one polymorphism in one population in an amplicon had a HWE p-value below 0.01. Twenty-seven polymorphisms had HWE p-values below this threshold in one population excluding the following promoters. Six promoters had multiple polymorphisms that failed HWE tests. Five promoters (*ABCF3*, *SLC22A5*, *ABCG1*, *ABCG4*, and *ABCB4*) had an excess of homozygosity, especially in the Chinese samples. One promoter (*SLCO6A1*) had an excess of heterozygotes. Polymorphisms in ABCF3 did not pass HWE equilibrium even though PCR and sequencing was preformed with two different primer sets. Eight percent of promoters were double scored; no major discrepancies were found and no additional polymorphisms were observed.

### Functional Characterization of Promoters

#### Construction of the membrane transporter promoter region

To construct the reporter plasmids containing the proximal promoter regions of the membrane transporters, we used Primer 3 (http://frodo.wi.mit.edu/cgi-bin/primer3/primer3_www.cgi) to design primers by inputting 500 bp of upstream and 100 bp downstream sequence relative to the predicted transcription start site (TSS) of the gene. These regions were amplified by a PCR of human genomic DNA using the touchdown PCR protocol [17] or other PCR protocols reported previously by our group [13], [14]. The primers used to construct the membrane transporter promoter regions are listed in Table S9. The amplified promoter fragments were digested with the appropriate restriction enzymes (New England Biolabs) and cloned into the reporter gene expression vector pGL-4 (Promega), which contains a Firefly luciferase reporter gene downstream from the multiple cloning site.

#### Cell culture, transient transfections, and luciferase activity assays

Similar protocols were used for determining the activity of ABC and SLC transporter promoters. For the ABC transporters, the constructs were transiently transfected into HepG2, PANC-1, HCT-116, JEG-3 and T98 cells, using a previously reported transfection protocol [17]. These cell lines were purchased from the American Type Culture Collection (Manassas, VA). A slightly modified protocol, described in detail in Tahara et al. and Yee et al., was used to transfect the SLC transporter proximal promoter constructs into HepG2, HCT-116 and ACHN cells. ACHN cells were purchased from the American Type Culture Collection (Manassas, VA) and the HepG2 and HCT-116 were supplied by the Cell Culture facility (University of California San Francisco). Briefly, 24 hours after transfection the cells were assayed for luciferase activity in a PE Wallac Luminometer^TM^ (Perkin Elmer) or GloMax 96 Microplate Luminometer (Promega) using the Dual-Glo^TM^ Luciferase Assay System (Promega) according to the manufacturer's instructions. For normalization of the data between cell types, the relative promoter activities were expressed as *log_2_ [(1+ Firefly luciferase/Renilla luciferase)/Average of the negative control]* [11].

### Association of Variants with Transporter Expression Levels

SNPs found both in our study populations and in the HapMap dataset (Phase II, release 23) were associated with transporter mRNA expression levels in lymphoblastoid cell lines derived from the 210 unrelated HapMap individuals [29]. Genotype data were downloaded from the HapMap website (http://www.hapmap.org), and normalized expression data from http://www.sanger.ac.uk/humgen/genevar/. This data has been deposited in the MIAME database with accession number GSE6536. Associations were calculated using PLINK v1.04 [42] (http://pngu.mgh.harvard.edu/purcell/plink/) for 46 common SNPs (MAF≥5%) that were found in both datasets, as well as for all common HapMap SNPs located within a ±50,000 bp range surrounding the same transporter genes. A moderate nominal significance level of *p*<0.01 was used to determine if significantly associated SNPs were found at higher frequency in the sequenced promoter regions compared to in the entire ±50,000 bp range.

### Population Genetic Parameters

Nucleotide diversity was estimated using the mutation parameter (θ; Eq. 1) and the average heterozygosity per site parameter (π; Eq. 2) (Tajima, 1989), assuming an infinite sites neutral mutation model:
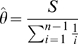
(1)

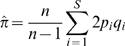
(2)where *S* is the number of segregating sites, *n* is the number of chromosomes analyzed and *p_i_* and *q_i_* are the frequencies of the non-ancestral and ancestral alleles, respectively. Both measures were normalized for the number of bases sequenced. Two different test statistics were used to assess deviations from the variation patterns expected under the neutral mutation model: Tajima's *D* [43] statistic was calculated as the normalized difference between the π and θ statistics, and Fay and Wu's *H* statistic [33], [44] was calculated as the difference between π and θ*_H_*:
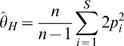
(3)


In the calculation of *H*, the chimpanzee allele (UCSC Genome Browser *Pan Troglodytes* March 2006 assembly) was used as the ancestral human allele. Variant sites were included in the analysis only if the chimpanzee allele matched one of the determined human alleles and the local (±5 bp surrounding the variant site) chimpanzee–human alignment was unambiguous. To assess statistical significance, the observed values of *D* and *H* were compared to the parameter distributions in simulated populations having the corresponding number of chromosomes and segregating sites. Ten thousand simulations were performed for each sequenced region using the *ms* software [45] under the conservative assumption of no recombination. The proportion of simulated *H* or *D* parameters with more extreme values than those observed corresponds to the hypothesis test *p*-value.

Nucleotide diversity was calculated for each transporter individually, as well as for various groups of genes (e.g., ABC transporters, SLC transporters, transporters highly expressed in the liver and/or the kidney, transporters with high or low promoter activity in *in vitro* assays). For each transporter/group of transporters, parameters were calculated for the proximal promoter region (+50 to −250 bp surrounding the transcription start site), the 5′ flanking sequence, and the entire sequenced regions.

To determine the distribution of genetic variation relative to the transcription start sites, θ and π were calculated in a 100 bp sliding window and plotted against the position of the window center (Fig. 2). The results were robust for changes in window size (20, 50, 100, and 200 bp windows were examined). The promoter regions of 5 genes (*ABCC2*, *SLC29A1*, *SLC28A2*, *SLC7A5*, and *SLC13A1*) were sequenced in a similar but separate set of individuals within the SOPHIE cohort. To enable comparisons within the same sample set, these transporters were not included in the calculations of population genetics statistics.

## Supporting Information

Table S1 (0.02 MB XLS)

Table S2 (0.14 MB XLS)

Table S3 (0.23 MB XLS)

Table S4 (0.09 MB XLS)

Table S5 (0.01 MB XLS)

Table S6 (0.01 MB XLS)

Table S7 (0.01 MB XLS)

Table S8 (0.02 MB XLS)

Table S9 (0.01 MB XLS)
